# Severe Tachycardia Associated with Psychotropic Medications in Psychiatric Inpatients: A Study of Hospital Medical Emergency Team Activation

**DOI:** 10.3390/jcm10071534

**Published:** 2021-04-06

**Authors:** Andy K. H. Lim, Meor Azraai, Jeanette H. Pham, Wenye F. Looi, Daniel Wirth, Ashley S. L. Ng, Umesh Babu, Bharat Saluja

**Affiliations:** 1 Department of General Medicine, Monash Health, Clayton, VIC 3168, Australia; meorazraai.ahmad@monashhealth.org (M.A.); jeanette.pham@monashhealth.org (J.H.P.); wenye.looi@monashhealth.org (W.F.L.); daniel.wirth@monashhealth.org (D.W.); angg0007@student.monash.edu (A.S.L.N.); 2 Department of Medicine, School of Clinical Sciences, Monash University, Clayton, VIC 3168, Australia; 3 Department of Psychiatry, Monash Health, Clayton, VIC 3168, Australia; umesh.babu@monashhealth.org (U.B.); bharat.saluja@monashhealth.org (B.S.)

**Keywords:** antipsychotic agents, neuroleptics, psychotropic drugs, psychiatry, drug side effects, tachycardia, heart rate, hospital medical emergency team, rapid response team

## Abstract

The use of antipsychotic medications is associated with side effects, but the occurrence of severe tachycardia (heart rate ≥ 130 per minute) is not well described. The aim of this study was to determine the frequency and strength of the association between antipsychotic use and severe tachycardia in an inpatient population of patients with mental illness, while considering factors which may contribute to tachycardia. We retrospectively analyzed data from 636 Medical Emergency Team (MET) calls occurring in 449 psychiatry inpatients in three metropolitan hospitals co-located with acute medical services, and used mixed-effects logistic regression to model the association between severe tachycardia and antipsychotic use. The median age of patients was 42 years and 39% had a diagnosis of schizophrenia or psychotic disorder. Among patients who experienced MET calls, the use of second-generation (atypical) antipsychotics was commonly encountered (70%), but the use of first-generation (conventional) antipsychotics was less prevalent (10%). Severe tachycardia was noted in 22% of all MET calls, and sinus tachycardia was the commonest cardiac rhythm. After adjusting for age, anticholinergic medication use, temperature >38 °C and hypoglycemia, and excluding patients with infection and venous thromboembolism, the odds ratio for severe tachycardia with antipsychotic medication use was 4.09 (95% CI: 1.64 to 10.2).

## 1. Introduction

In patients with schizophrenia and other psychotic disorders, antipsychotic medications relieve hallucinations and delusions, improve thought disorder, behavior and quality of life, and ultimately allow patients to function in society [1]. Antipsychotics can be used as adjunct treatment in severe anxiety and mood disorders, and in patients with dementia with behavioral and psychological symptoms. They are broadly divided into the first-generation antipsychotics (used since the 1950s) such as haloperidol, chlorpromazine, flupentixol, fluphenazine, trifluoperazine, and pericyazine; and second-generation or atypical antipsychotics (used since the 1990s) such as olanzapine, risperidone, quetiapine, amisulpride, aripiprazole, paliperidone, and clozapine.

Antipsychotic medications are associated with multiple adverse effects. Among these, several mechanisms may contribute to tachycardia, such as the anticholinergic properties of antipsychotics, postural hypotension due to α1-adrenoreceptor blockade, and indirect effects such as myocarditis and neuroleptic malignant syndrome [2,3]. Most cases of tachycardia are mild and can be managed with dose adjustment and good fluid intake. However, the occurrence of severe or clinically serious tachycardia is not well described, with limited data on the frequency and strength of the association.

In Australia, New Zealand, and other countries, a rapid response team such as the hospital Medical Emergency Team (MET) is activated upon recognition of worsening vital signs or clinical concern in a hospitalized patient. The MET call brings critical care expertise to the bedside to evaluate and stabilize deteriorating patients [4]. In psychiatric facilities co-located with acute medical services, MET calls for tachycardia are relatively common and a number of these may be due to the adverse effects of antipsychotic medications. Given the medical complexity of some patients with mental illness, other factors may confound the association between antipsychotic medications and severe tachycardia requiring a rapid response team review.

The aims of this study were to determine the frequency and strength of the association between antipsychotic medication use and severe tachycardia at MET calls in an inpatient population of patients with mental illness, while considering other factors which may contribute to tachycardia in a hospitalized patient.

## 2. Methods

### 2.1. Study Design and Setting

We conducted a cross-sectional study of all patients who had a MET call during their admission to a Monash Health psychiatric facility from January 2015 to January 2020. The Monash Health hospital network is in the state of Victoria, Australia and provides healthcare services to the south-east region of the city of Melbourne. Within the network, three hospitals (Monash Medical Centre, Clayton; Dandenong Hospital, Dandenong; Casey Hospital, Berwick) have inpatient psychiatric services that are co-located with acute medical services, which provide 24-h onsite MET availability. The inpatient capacity across the three sites is 188 beds, comprising general adult psychiatry, mother–baby unit, aged psychiatry (≥65 years), and a secured extended care unit (SECU) for patients requiring medium to longer term treatment for unremitting and severe symptoms.

### 2.2. Participants and Ethics Approval

We searched our central electronic incident reporting system (RISKMAN) for adult psychiatry inpatients ≥18 years who experienced a MET call during the study period at any of the three sites. We excluded cases with inadequate or missing documentation of the MET call, any duplicated entries and patients receiving day treatment who were incorrectly identified as an inpatient, and visitors to the ward. We also excluded patients returning from a leave of absence within the previous 24 h, as these patients may have MET calls and tachycardia related to illicit drug use.

The Monash Health Human Research Ethics Committee approved this study as a quality improvement initiative and waived the need for patient consent (RES-20-0000281Q-63108).

### 2.3. Medical Emergency Team

For this study, MET calls and MET calls which progress to Code Blue (cardiopulmonary arrest) were considered together. Our hospital guidelines mandate MET activation if any one or more of the following criteria are fulfilled: (1) respiratory distress, (2) concern of the airway, (3) respiratory rate >30/min or <6/min, (4) oxygen saturation <90%, (5) systolic blood pressure <90 mm Hg, (6) heart rate >130/min or <40/min, or (7) a sudden decrease in conscious state. Trigger zones are color coded in the observation charts and electronic record. Discretionary MET calls were also allowed for a broader criterion of clinical concern, even if vital signs have not breached the threshold for mandatory MET activation. Such clinical concerns may include, but not limited to chest pain, poor urine output, hypoglycemia, trauma, and self-harm.

### 2.4. Study Outcome and Variable Definitions

The main study outcome was severe tachycardia, as defined by the MET call criterion of a heart rate ≥130 per minute. The main factor of interest was antipsychotic medication use, which was grouped into two broad categories: first-generation or conventional antipsychotics, and second-generation or atypical antipsychotics. A comprehensive review of the drug charts was undertaken to identify all psychotropic medications prescribed and any other medication which may be associated with alterations in heart rate. We grouped the other psychotropic medication into categories of antidepressants, mood-stabilizers, and benzodiazepines. We specifically collected data on the use of anticholinergic agents to manage the extrapyramidal side-effects of antipsychotic medications as these can also contribute to tachycardia.

From the medical records, we collected demographic information and determined the primary psychiatric diagnosis related to admission. We identified baseline independent variables which could confound the association between antipsychotic medications and tachycardia. They included age, sex, body mass index (BMI) categories (ideal, underweight, or obese), smoking, alcoholism, illicit drug use, recent electroconvulsive treatment (ECT) and the comorbidities of diabetes, a composite of cardiovascular disease (coronary artery disease, heart failure, stroke), and a composite of chronic lung disease (asthma, chronic obstructive pulmonary disease, and interstitial lung disease).

From the pathology system, we collected data on the serum potassium and magnesium levels measured during the MET call or within 48 h of the MET call. Biochemistry results beyond 48 h of the MET call were considered unreliable for analysis. The laboratory reference range for normal serum potassium was 3.5 to 5.2 mmol/L, and the reference range for serum magnesium was 0.70 to 1.10 mmol/L.

We examined the RISKMAN entry and medical record to determine the trigger for the MET call, the observations and assessment of the patient during the MET call (vital signs, Glasgow Coma Scale (GCS), oxygen saturation and temperature), and reviewed the patient’s electrocardiogram (ECG) to determine the underlying rhythm associated with the tachycardia. Finally, we examined the final diagnosis associated with the MET call for additional confounders of tachycardia.

### 2.5. Statistics

In the descriptive analysis, we report the mean and standard deviation (SD) for data which were normally distributed. For skewed data, we report the median and interquartile range (IQR). We examined the association between two categorical variables using a χ^2^ analysis or Fisher’s exact test. As more than one MET call per patient was possible, we used mixed-effects logistic regression (individual patients as clusters) to test the association between tachycardia and antipsychotic use, and adjusting for several confounders. For variable selection into the multivariable model, we used the purposeful selection method. All variables with a *p* < 0.25 from the univariable analysis were entered into the multivariable model, followed by backwards elimination of variables with the least statistical significance. Eliminated variables were checked for confounding by assessing the change in the estimated coefficient for antipsychotic use. A change of >10% was considered evidence of confounding. Variables with a *p* < 0.05 and confounding variables were retained. Fractional polynomials were used to confirm the linearity of the logit for continuous variables in the model. We checked for collinearity by examining the variance inflation factor, and for statistical interaction between the main effect variables at a 1% level. Akaike and Bayesian information criteria were used to compare different possible models. In the final multivariable model, we examined the calibration plot to assess model fit, and we report the cluster-specific (conditional) adjusted odds ratios for antipsychotic medication use. Analysis was performed with STATA v16.1. (StataCorp, College Station, TX, USA). A *p* < 0.05 was considered statistically significant.

## 3. Results

### 3.1. Patient Characteristics

**Figure 1:**
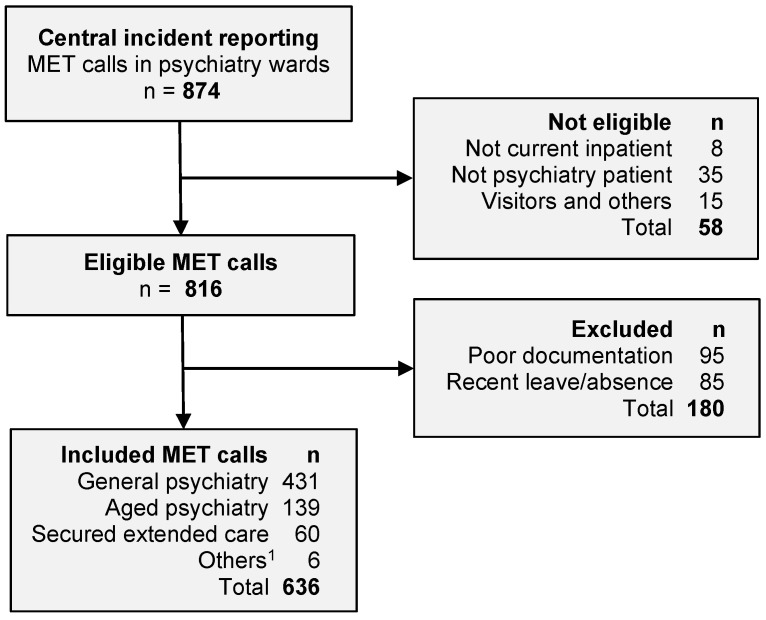
Study flow diagram of eligible patients, reasons for exclusion, and number of included patients by location. ^1^ Includes eating disorders and mother–baby unit. Abbreviation: MET, Medical Emergency Team.

A flow diagram of the selection of eligible patients and the reasons for exclusion is shown in Figure 1. The study included 449 individual patients who experienced a total of 636 MET calls. The characteristics of the patients are summarized in Table 1. The study population included acute psychiatric admissions and longer-term residents located in the aged psychiatry unit and the secured extended care unit (SECU). Thus, the inpatient length of stay varied from 2 days to more than one year. Overall, 46% of admissions lasted less than 30 days and 76% were less than 90 days. Less than 14% of patients resided for more than 6 months.

At the time of the MET calls, the prevalence of hypokalemia and hypomagnesemia were 3.9% and 8.8%, respectively (Table 1). The distribution of these electrolyte levels and the proportion of patients with levels outside the reference ranges are shown in Supplementary Figure S1 (potassium) and Supplementary Figure S2 (magnesium), with patients stratified by the outcome status. The mean serum potassium of patients with severe tachycardia was 0.08 mmol/L lower than those without, which was not significant (*t*_594_ = 1.48, *p* = 0.14). The mean serum magnesium of patients with severe tachycardia was 0.01 mmol/L lower than those without, which was also not significant (*t*_510_ = 0.87, *p* = 0.39).

**Figure 2:**
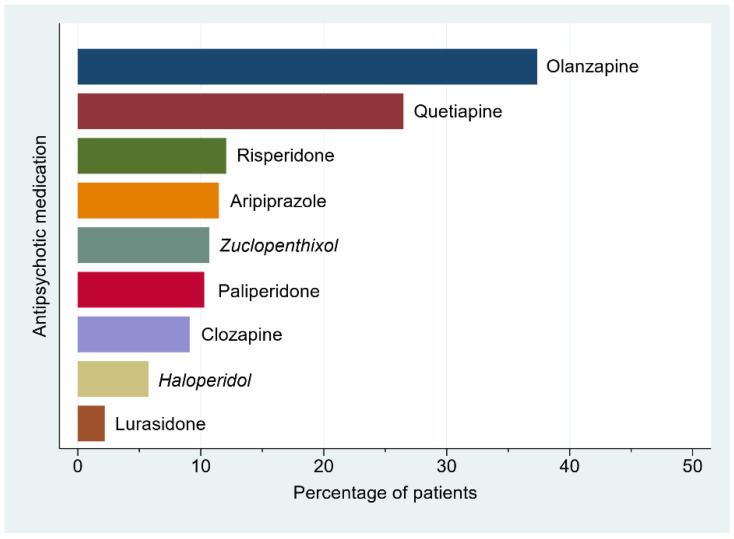
Distribution of inpatient antipsychotic medication use (*n* = 506). First-generation antipsychotics are italicized. Antipsychotics with <1% frequency of use are not displayed, which included the first-generation antipsychotics: chlorpromazine, flupentixol, pericyazine, and droperidol; and the second-generation antipsychotics: brexpiprazole, asenapine, and amisulpride.

Patients with schizophrenia and other psychotic disorders comprised a large proportion of the inpatient population, and consequently, antipsychotic medications, particularly the second-generation antipsychotics, were the most frequent class of psychotropic medication used (Table 2 and Figure 2).

### 3.2. Tachycardia

The top three triggers for a MET call were a drop in GCS or seizure (39.6%), tachycardia (16.8%) and hypotension (16.7%). The other triggers for a MET call included hypoxia (6.4%), tachypnea (2.0%), bradycardia (1.7%), and the rest were for clinical concern such as chest pain, hypoglycemia, and self-harm. On clinical assessment, 137 of 636 (21.5%) of MET calls were associated with tachycardia and a heart rate of ≥130 per min regardless of the MET call trigger. An ECG was performed during 383 (60.2%) of MET calls. Of the 383 ECGs examined, 190 (49.6%) were completely normal, and a detailed description of the abnormal ECGs is presented in Table 3. With regards to patients with severe tachycardia (≥130 per min) where an ECG was available (109 of 137 MET calls), sinus tachycardia was the most frequent rhythm on ECG (88.1%), followed by atrial fibrillation with rapid ventricular rate (7.3%), supraventricular tachycardia (3.7%), and ventricular tachycardia (0.9%).

### 3.3. Factors Associated with Tachycardia

The results of the univariable logistic regression analyses are summarized in Table 4. For the main factor of interest, we found a significant association between antipsychotic medication use and tachycardia. As a binary class effect, the univariable odds ratio for antipsychotic medication use was 4.71 (95% CI: 1.83 to 12.1). When parameterized as nominal variables, the first-generation antipsychotics demonstrated a higher univariable odds ratio compared to the second-generation antipsychotics (Table 4).

In the multivariable model (Table 5), there was evidence that age, temperature >38 °C, and anticholinergic medication use were associated with tachycardia. Although hypoglycemia and benzodiazepine use were not statistically significant at the 5% level in the multivariable model, these variables changed the estimated coefficient for antipsychotics by ≥10% when omitted from the model. Model 1 (antipsychotics as a binary variable) provided a single odds ratio for the class effect. Model 2 (antipsychotics as a nominal variable) may be more informative and showed a higher odds ratio for tachycardia with first- compared to second-generation antipsychotics.

In the sensitivity analysis, excluding MET calls associated with patients diagnosed with venous thromboembolism or infection (Model 3), the estimated coefficient for antipsychotics changed by 12%. By excluding these patients with reasons for tachycardia, benzodiazepine use was not statistically significant (*p* = 0.20) and only changed the estimated coefficient by 7% if dropped from the model, but the effect of hypoglycemia on the coefficient for antipsychotics remained greater than 10%. In Model 4, benzodiazepine use was dropped, and the Akaike and Bayesian information criteria suggested that this model provided the best fit for our data and is our preferred model for inference. In this model, there was no evidence of statistical interaction or multicollinearity among the included variables. The model testing and goodness-of-fit assessment is presented in Supplementary Figure S3. We did not report model discrimination as the analysis was not intended for prediction.

### 3.4. Outcomes

In patients with severe tachycardia at MET calls, 68 of 137 (50%) were transferred to an acute medical ward. This was identical to the 248 of 499 (50%) of patients without tachycardia who were transferred to an acute medical ward after a MET call. Hence, tachycardia per se did not appear to be a factor associated with transfer. In patients who were transferred, the length of stay in the acute medical ward was not different in tachycardic (median, 3 days; IQR, 1 to 5 days) vs. non-tachycardic patients (median, 3 days; IQR, 2 to 6 days), with a *p* = 0.33. There was also no difference in the odds ratio for in-hospital mortality in tachycardic vs. non-tachycardic patients (OR 0.55, 95% CI: 0.08 to 3.75, *p* = 0.54).

## 4. Discussion

In this study of MET calls in an inpatient psychiatric population, we found a positive association between the use of antipsychotic medications and severe tachycardia (heart rate ≥130 per min). A patient in a psychiatric ward who was treated with any antipsychotic medication increased his or her odds of experiencing severe tachycardia and a MET call by 4.1 times the odds if he or she did not use antipsychotic medications, holding age, temperature >38 °C, anticholinergic medication use, and hypoglycemia constant. The odds of tachycardia were even higher if he or she was treated with the first- generation compared to second-generation antipsychotics, although this difference was small and the clinical relevance was unclear.

Cardiovascular side-effects of antipsychotic medications are well known. Sudden death and ventricular arrythmias associated with prolongation of the QT interval is the most dreaded [5,6]. However, we demonstrated that supraventricular arrhythmias were more prevalent than ventricular arrhythmias. The majority of supraventricular arrhythmias were episodes of sinus tachycardia, but we were also concerned that antipsychotic medication use may be associated with atrial fibrillation (OR 3.82, 95% CI: 0.50 to 29.3, *p* = 0.20). We did not have statistical power to confirm this association, and it was an a posteriori consideration. However, a previous population-based study reported that the use of antipsychotics was associated with a 17% increased risk of atrial fibrillation compared to non-use, and patients at highest risk were those with hypertension, diabetes, and ischemic heart disease [7].

The association between antipsychotics and severe tachycardia in our study raises questions regarding the long-term risk of tachycardia-induced cardiomyopathy (TIC), where prolonged tachycardia induces structural and clinical features of heart failure with reduced ejection fraction. Continuous ambulatory monitoring studies have demonstrated that antipsychotic medication use can be associated with persistent tachycardia [8]. In a pooled analysis of clinical trials, patients treated with antipsychotics had a 2.4-times (95% CI: 1.5 to 36 times) higher odds of tachycardia and 1.7-times (95% CI: 1.2 to 2.4 times) higher odds of heart failure compared to patients treated with placebo [9].

TIC may be a phenomenon related to rate-related ventricular dysfunction, with depletion of energy stores and ischemia, oxidative stress, and neurohormonal activation leading to myocardial fibrosis and cardiac structural changes [10,11]. Catheter ablation studies convincingly show reversal of TIC in 88% to 97% of patients with successful ablation [12,13]. Therefore, TIC is preventable. Most cases of TIC are attributable to several supraventricular arrhythmias, but any incessant or frequent paroxysmal tachycardia can result in TIC if severe and prolonged. Sustained sinus tachycardia has been mentioned as a cause of TIC in one review [11], but not another [10]. Studies specifically examining antipsychotic-related tachycardia and TIC are lacking. Inference may be drawn from a handful of case reports of patients with inappropriate sinus tachycardia (IST) or prolonged sinus tachycardia with trauma who developed TIC [14,15,16]. For now, one longitudinal study examining cardiac structure in patients with IST did not detect evidence of heart decompensation after an average follow-up of almost 5 years [17].

In our study, there was a small difference in the odds ratio for severe tachycardia and MET calls between the first- and second-generation antipsychotics. Although we assumed a class effect in our analysis, other studies have suggested that individual antipsychotics vary in their side-effect profile (even within the same class of second-generation antipsychotics) and demonstrate a dose-dependent effect, including their effect on heart rate [18,19]. Other reports have suggested that clozapine is more potent at inducing tachycardia than other antipsychotics due to the potent anticholinergic properties [19,20,21]. Ultimately, all antipsychotics have the potential to contribute to the severe tachycardia associated with in inpatient MET call.

Hypokalemia is associated with abnormal intracellular calcium and sodium metabolism, which can trigger arrhythmias such as ventricular tachycardia/fibrillation, atrial fibrillation, and atrial flutter [22,23]. In our study, the odds of severe tachycardia were higher in patients with hypokalemia compared to normokalemia, but not statistically significant. One reason may be the low prevalence of hypokalemia in this population and the study was underpowered to detect a difference. Hypomagnesemia has been associated with a variety of ventricular and supraventricular arrhythmias [24]. However, there was no indication in our study that hypomagnesemia was associated with higher odds of severe tachycardia even though the prevalence of hypomagnesemia was higher than hypokalemia. Patients with myocardial infarction and heart failure are more susceptible to arrhythmias associated with hypomagnesemia [24,25]. Our patients were mostly young and had a low cardiovascular disease burden. Overall, we do not believe that electrolyte abnormalities contributed to severe tachycardia in our patients, where the predominant rhythm was sinus and supraventricular tachycardias.

One strength of our study was the inclusion of multiple sites and a range of psychiatric diagnoses, which improves the generalizability of the results. We used a multivariable logistic regression model to account for confounders, and used a mixed effects model to account for the influence of random effects (of recurrent MET calls) on the parameter estimates. We also collected data on relevant medications which may protect against severe tachycardia or act synergistically with antipsychotics to worsen tachycardia. The main limitation of our study was the cross-sectional design which limits a direct causal inference. We could not factor in antipsychotic medication dosage, which, although relevant, was difficult retrospectively, given titration of doses at the clinician’s discretion, use of long-acting preparations, and the irregular use of *prn* (as needed) breakthrough or supplemental doses. As a cross-sectional study, we cannot prove that tachycardia was sustained or severe and a longitudinal study would be ideal to address this. There were missing observations in our data for potassium and magnesium levels, which may have biased our analysis. A prospective cohort study of patients initiating antipsychotics after a period of baseline monitoring could avoid issues of missing observations and allow accurate quantification of antipsychotic medication exposure, and thus provide better quality evidence for a causal inference. Finally, as we only studied an inpatient population, the results should not be generalized to patients in the community or ambulatory care.

## 5. Conclusions

Our study confirms a strong association between antipsychotics and severe tachycardia in psychiatric inpatients. The potential for adverse cardiac outcomes in the medium- to long-term due to severe tachycardia is unknown and needs to be further studied to assist in future risk–benefits discussion.

## Figures and Tables

**Table 1:** Characteristics of patients experiencing Medical Emergency Team calls for severe tachycardia.

Patient Characteristic	All MET Calls *n* = 636	No Tachycardia *n* = 499	Tachycardia *n* = 137
Age, median (IQR), years	42 (30–61)	45 (31–64)	36 (28–46)
Male, *n* (%)	324 (50.9)	260 (52.1)	64 (46.7)
Body mass index <18 kg/m^2^, *n* (%)	38 (6.0)	31 (6.2)	7 (5.1)
Diabetes mellitus, *n* (%)	138 (21.7)	112 (22.4)	26 (19.0)
Cardiovascular disease, *n* (%) ^1^	85 (13.4)	73 (14.6)	12 (8.8)
Coronary artery disease	52 (8.2)	46 (9.2)	6 (4.4)
Heart failure	25 (3.9)	19 (3.8)	6 (4.4)
Stroke	28 (4.4)	24 (4.8)	4 (2.9)
Atrial fibrillation, *n* (%)	16 (2.5)	8 (1.6)	8 (5.8)
Chronic	12 (1.9)	6 (1.2)	6 (4.4)
Acute onset	4 (0.6)	2 (0.4)	2 (1.5)
Chronic lung disease, *n* (%)	96 (15.1)	71 (14.2)	25 (18.3)
Smoking, *n* (%)	296 (46.5)	229 (45.9)	67 (48.9)
Excessive alcohol, *n* (%)	144 (22.6)	106 (21.2)	38 (27.7)
Illicit drug use, *n* (%)	255 (40.1)	189 (37.9)	66 (48.2)
Recent ECT, *n* (%)	40 (6.3)	31 (6.2)	9 (6.6)
Potassium, mean (SD), mmol/L ^2^	4.19 (0.52)	4.20 (0.52)	4.13 (0.52)
Hypokalemia, *n* (%) ^2^	23 (3.9)	15 (3.2)	8 (6.1)
Hyperkalemia, *n* (%) ^2^	16 (2.7)	14 (3.0)	2 (1.5)
Magnesium, mean (SD), mmol/L ^3^	0.83 (0.10)	0.83 (0.10)	0.82 (0.10)
Hypomagnesemia, *n* (%) ^3^	45 (8.8)	37 (9.5)	8 (6.6)
Hypermagnesemia, *n* (%) ^3^	4 (1.0)	0 (0)	4 (0.8)
Primary psychiatric diagnosis:			
Schizophrenia/psychotic, *n* (%)	249 (39.2)	170 (34.1)	79 (57.7)
Anxiety/depression, *n* (%)	117 (18.4)	89 (17.8)	28 (20.4)
BPSD, *n* (%)	64 (10.1)	60 (12.0)	4 (2.9)
Bipolar disorder, *n* (%)	60 (9.4)	51 (10.2)	9 (6.6)
Alcohol & substance misuse, *n* (%)	55 (8.6)	50 (10.0)	5 (3.6)
Stress & adjustment disorder, *n* (%)	18 (2.8)	16 (3.2)	2 (1.5)
Other disorders, *n* (%)	73 (11.5)	63 (12.6)	10 (7.3)

^1^ Composite of coronary artery disease, heart failure and stroke. ^2^ Missing observations, 40 (6.3%). ^3^ Missing observations, 124 (19.5%). Abbreviations: MET, Medical Emergency Team; ECT, electroconvulsive treatment; BPSD, Behavioral and psychological symptoms of dementia.

**Table 2:** Psychotropic and other medications used at the time of the Medical Emergency Team call.

Medications, *n* (%)	All MET Calls *n* = 636	No Tachycardia *n* = 499	Tachycardia *n* = 137
Antipsychotics:			
Conventional (first-generation)	63 (9.9)	44 (8.8)	19 (13.9)
Atypical (second-generation)	443 (69.7)	338 (67.7)	105 (76.6)
Antidepressants:			
SSRI or SNRI	206 (32.4)	170 (34.1)	36 (26.3)
Tricyclic antidepressants	38 (6.0)	31 (6.2)	7 (5.1)
Combination & others ^1^	25 (3.9)	16 (3.2)	9 (6.6)
Mood stabilizers:			
Valproate	126 (19.8)	100 (20.0)	26 (19.0)
Lithium	28 (4.4)	22 (4.4)	6 (4.4)
Others ^2^	32 (5.0)	30 (4.7)	2 (1.5)
Other medications:			
Benzodiazepines	398 (62.6)	320 (64.1)	78 (56.9)
Anticholinergics	51 (8.0)	32 (6.4)	19 (13.9)
Antiplatelets	71 (11.2)	64 (12.8)	7 (5.1)
Anticoagulation	44 (6.9)	36 (7.2)	8 (5.8)
Beta-blockers	104 (16.4)	82 (16.4)	22 (16.1)
Renin-angiotensin system inhibitor	78 (12.3)	70 (14.0)	8 (5.8)
Calcium channel blocker	24 (3.8)	24 (4.8)	0 (0)
Diuretics	37 (5.8)	29 (5.8)	7 (5.1)
Oral hypoglycemic or insulin	102 (16.0)	83 (16.6)	20 (14.6)
Inhaled bronchodilators	86 (13.5)	65 (13.0)	21 (15.3)
Opioid analgesics	104 (16.4)	89 (17.8)	15 (11.0)

^1^ Includes norepinephrine reuptake inhibitors and monoamine oxidase inhibitors. ^2^ Includes carbamazepine, lamotrigine, and combination of two mood stabilizers. Abbreviations: MET, Medical Emergency Team; SSRI, selective serotonin reuptake inhibitor; SNRI, selective serotonin-norepinephrine reuptake inhibitor.

**Table 3:** Abnormal electrocardiogram findings during MET calls (*n* = 193).

Electrocardiogram Findings	Number	Percent
Sinus tachycardia	117	60.6
Prolonged QTc interval	21	10.9
Atrial fibrillation	16	8.3
Conduction or T-wave abnormality	16	8.3
Sinus bradycardia	14	7.3
Supraventricular tachycardia	6	3.1
Other	3	1.6

**Table 4:** Univariable logistic regression analysis of tachycardia.

Variable	Odds Ratio (95% CI)	*p*-Value
Age, per 10 years	0.70 (0.58–0.84)	<0.001
Female	0.63 (0.33–1.22)	0.173
Body mass index:		
18 to 30 kg/m^2^	1.00 (reference)	0.950
<18 kg/m^2^	1.07 (0.27–4.31)	
>30 kg/m^2^	1.15 (0.48–2.77)	
Diabetes mellitus	0.83 (0.37–1.86)	0.645
Cardiovascular disease	0.42 (0.14–1.13)	0.085
Chronic lung disease	1.59 (0.69–3.68)	0.280
Cigarette smoking	1.18 (0.64–2.16)	0.600
Excessive alcohol intake	1.27 (0.87–1.85)	0.217
History of illicit drug use	1.85 (1.00–3.44)	0.051
Recent electroconvulsive treatment	1.46 (0.73–2.91)	0.283
Antipsychotics vs. none	4.71 (1.83–12.1)	0.001
Antipsychotics by class:		
None	1.00 (reference)	0.004
Conventional (first-generation)	7.40 (2.09–26.2)	
Atypical (second-generation)	4.42 (1.71–11.4)	
Antidepressants	1.05 (0.84–1.32)	0.651
Mood stabilizers	0.90 (0.75–1.09)	0.289
Benzodiazepines	0.64 (0.35–1.17)	0.149
Anticholinergics	4.09 (1.42–11.8)	0.009
Beta-blockers	1.15 (0.49–2.70)	0.739
Inhaled bronchodilators	1.06 (0.86–1.30)	0.584
Temperature >38 °C	5.96 (2.19–16.2)	<0.001
Diagnosis of infection	1.90 (0.79–4.56)	0.151
Diagnosis of dehydration	0.88 (0.31–2.45)	0.789
Venous thromboembolism	1.14 (0.33–3.91)	0.836
Hypoglycemia	0.23 (0.05–1.05)	0.059
Hypokalemia	2.74 (0.61–12.3)	0.187
Hypomagnesemia	0.60 (0.17–2.14)	0.433
Oxygen saturation <90%	0.64 (0.23–1.75)	0.380

**Table 5:** Models of a cluster-specific random effects multivariable logistic regression of severe tachycardia on antipsychotic medication adjusted for confounders.

Model	Specification	OR (95% CI)	ICC (95% CI)
1	Main effects model ^1^	4.98 (2.19–12.8)	0.39 (0.19–0.63)
2	Antipsychotics as nominal variable: ^1^		
	No antipsychotics	1.00 (reference)	0.38 (0.18–0.63)
	Conventional	6.54 (2.04–20.9)	
	Atypical	5.01 (2.05–12.2)	
3	Model 1 with VTE and infection excluded ^2^	4.40 (1.75–11.0)	0.36 (0.16–0.63)
4	Model 3 with benzodiazepine dropped	4.09 (1.64–10.2)	0.37 (0.17–0.63)

^1^ Adjusted for age, anticholinergic use, benzodiazepine use, temperature >38 °C and hypoglycemia. ^2^ Venous thromboembolism (VTE), *n* = 38; infection, *n* = 68. Notes: Random effects statistically significant in all models (*p* < 0.01) based on likelihood ratio tests. Abbreviations: OR, odds ratio; ICC, intracluster correlation.

## Data Availability

The data presented in this study are available on reasonable request from the corresponding author, subject to approval by the Monash Health research directorate.

## Supplementary Materials

The following are available online at https://www.mdpi.com/article/10.3390/jcm10071534/s1, Figure S1: Distribution of serum potassium levels stratified by the primary outcome; Figure S2: Distribution of serum magnesium levels stratified by the primary outcome. Figure S3: Multivariable model diagnostic testing and fit.
