# Supplementary material for: Severe Tachycardia Associated with Psychotropic Medications in Psychiatric Inpatients: A Study of Hospital Medical Emergency Team Activation
Source: J Clin Med. 2021 Apr 6;10(7):1534. doi: 10.3390/jcm10071534 (PMC8038822; doi:10.3390/jcm10071534)
Supplement: Supplementary file 1 [file jcm-10-01534-s001.pdf]

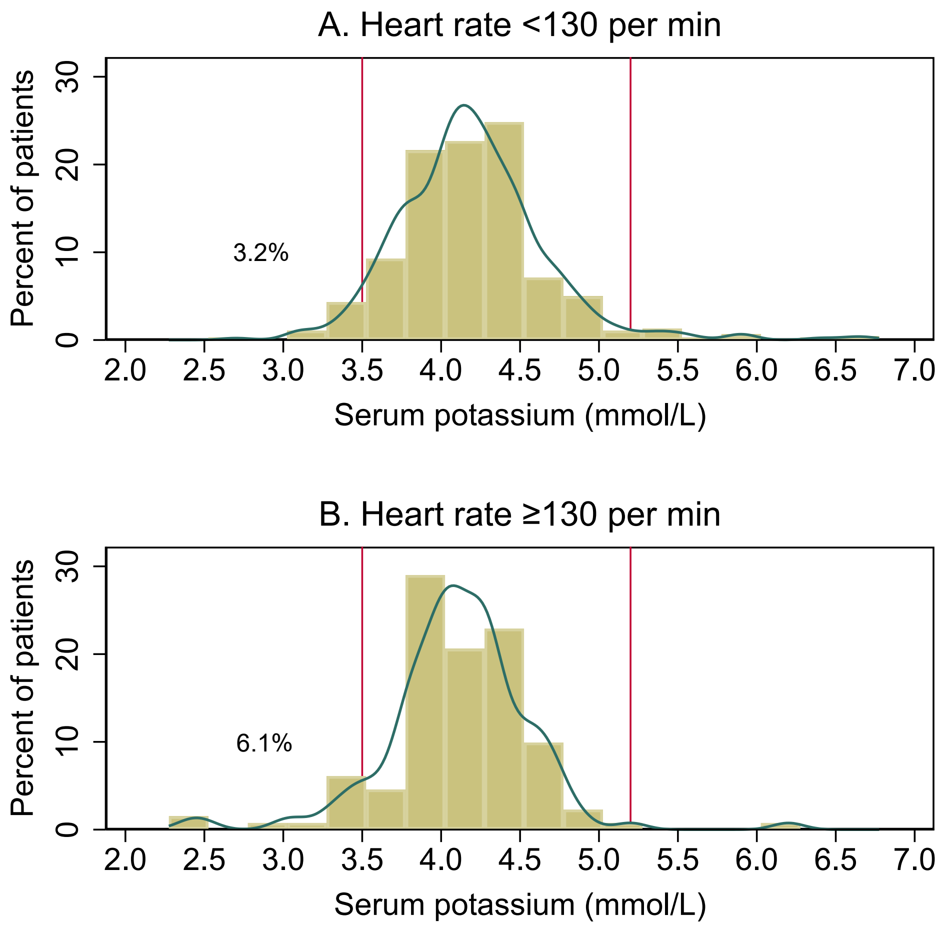

Figure S1. Histogram with kernel density curve (green) showing distribution of serum potassium levels performed at the time of Medical Emergency Team activation, stratified by the primary outcome of severe tachycardia. Data was available for 94% of patients (*n*=596). Panel A (top) demonstrates the distribution of serum potassium levels among patients who did not experience severe tachycardia, compared to patients who experienced severe tachycardia (Panel B, bottom). The vertical red lines represent the lower and upper limits of the normal laboratory reference range for serum potassium, and the percentage displayed represent the proportion of patients who lie below the normal reference range. The small differences in the mean serum potassium and the percentage of patients with hypokalemia between the two groups were not statistically significant.

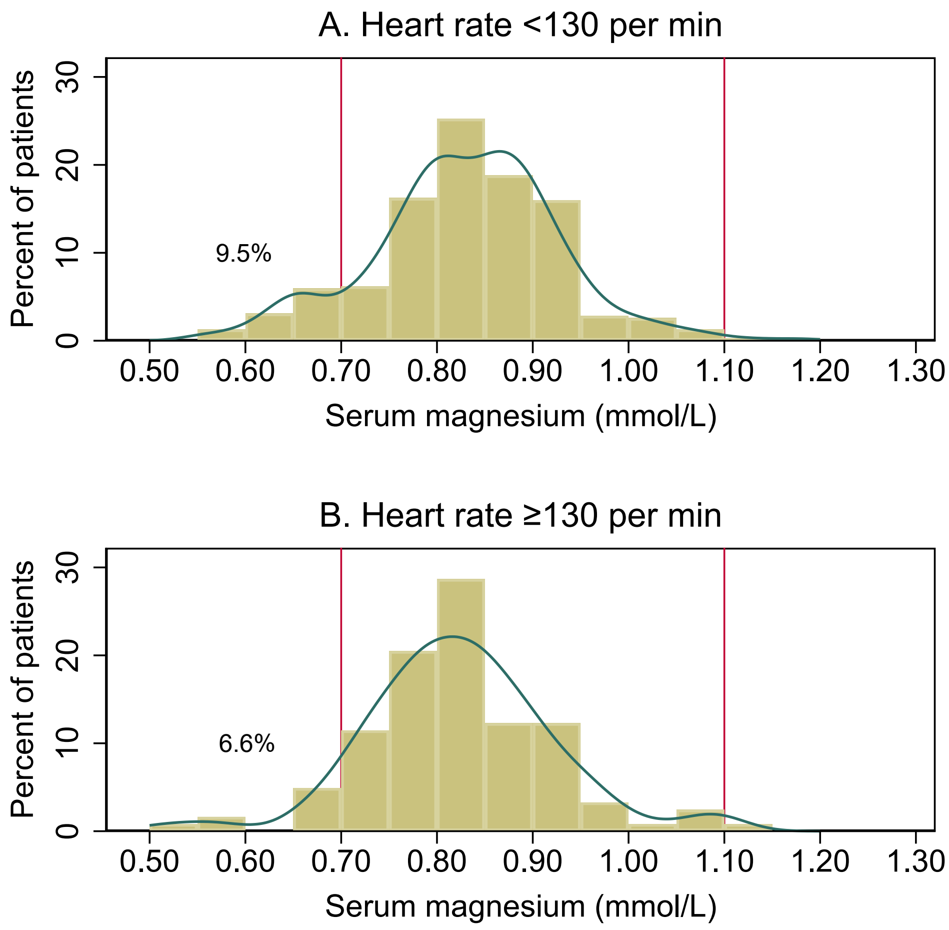

Figure S2. Histogram with kernel density curve (green) showing **d**istribution of serum magnesium levels performed at the time of Medical Emergency Team activation, stratified by the primary outcome of severe tachycardia. Data was available in 81% of all patients (*n*=512). Panel A (top) demonstrates the distribution of serum magnesium levels among patients who did not experience severe tachycardia, compared to patients who experienced severe tachycardia (Panel B, bottom). The vertical red lines represent the lower and upper limits of the normal laboratory reference range for serum magnesium, and the percentage displayed represent the proportion of patients who lie below the normal reference range. The small differences in the mean serum magnesium and the percentage of patients with hypomagnesemia between the two groups were not statistically significant.

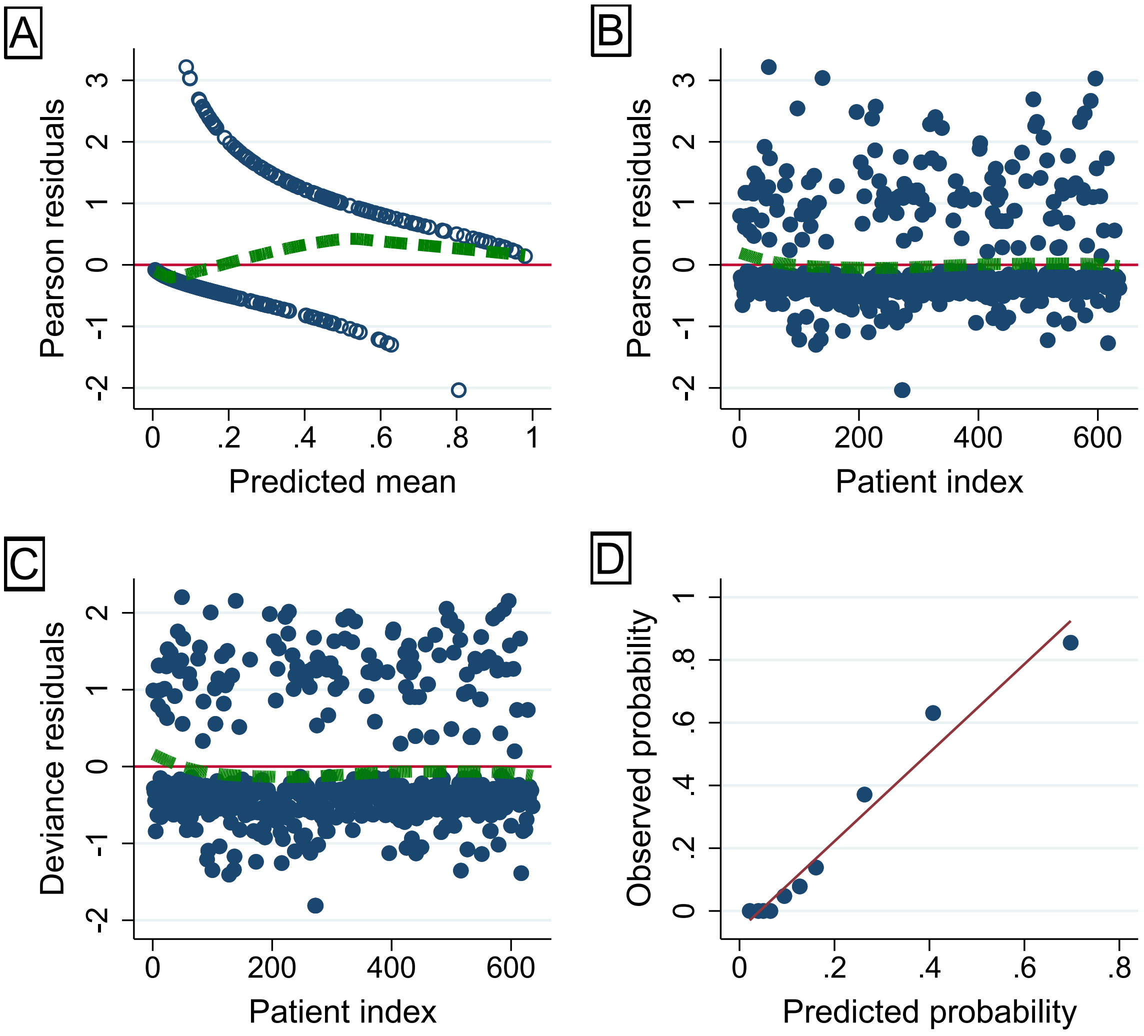

Figure S3. Multivariable model diagnostic testing and fit. The plot of Pearson residuals against the predicted values (**A**) and the patient index (**B**) do not demonstrate any outliers. The plot of the deviance residuals against the patient index (**C**) also supported the absence of outliers. The calibration plot of the predicted versus observed probabilities of severe tachycardia by deciles of risk (**D**) indicates a reasonable fit of the regression model for the data. Lowess curves for the residual plots for (**A)** to (**C**) in green (thick, dashed line) showed that overall, the residuals are centred around zero.
